# The importance of disability as a health issue for mid-life women

**DOI:** 10.1186/s40695-015-0011-x

**Published:** 2015-10-14

**Authors:** Carrie A. Karvonen-Gutierrez

**Affiliations:** Department of Epidemiology, University of Michigan School of Public Health, 1415 Washington Heights, Room 6618, Ann Arbor, MI 48109 USA

**Keywords:** Women, Mid-life, Middle age, Physical functioning, Functional limitations, Disability

## Abstract

Data suggest that disability prevalence among mid-aged populations is increasing in recent years; current prevalence estimates for mid-aged adults range from 20 to 40 %. The World Health Organization’s International Classification of Functioning (ICF) has provided a multi-dimensional biopsychosocial model to understand disability that is highly relevant to mid-aged populations. Under the ICF framework, mid-aged women experience high levels of work, non-work, and mobility-associated disability but very little difficulty with self care. Despite the high prevalence, evidence suggests that there is a large proportion of non-chronic disability and that mid-aged women can both worsen and improve their functioning. Thus, the mid-life period may represent a critical window during which interventions to improve disability may be most efficacious for the improvement of current and future functioning. Interventions that are initiated during the mid-life are highly relevant as a strategy to reduce disability during this life stage and prevent or forestall the onset of late life disability. Targets for intervention include improvement of depressive symptoms and increasing physical activity levels, both of which have shown to be efficacious in older populations and are correlates of mid-life functioning and disability.

## Introduction

Our world is in the midst of an epidemiologic transition whereby the burden of non-communicable diseases has now surpassed communicable diseases and injury as the leading cause of death and illness worldwide [1]. Globally, the rise in prevalence of obesity and adverse health behaviors including smoking, poor diet, and physical inactivity in concert with the overall rise in life expectancy has led to the exponential increase in chronic disease prevalence and multi-morbidity. Individuals are being diagnosed with chronic conditions earlier and have a greater number and more severe chronic conditions than ever before [2, 3]. In the United States, the average number of chronic conditions among midlife adults is increasing; the number of midlife adults with three or more chronic conditions increased by 9.7 % between 1996 and 2005 [4]. Thus, the burden of chronic disease and its effects on functioning and health is the major public health challenge of the 21st century.

The increase in chronic disease prevalence and severity is concerning because chronic diseases are the leading cause of disability in the United States [5] and globally [6], so it is expected that there will be a concomitant rise in disability. Current estimates from the World Health Survey and the Global Burden of Disease indicate that more than 1 billion people in the world (based upon 2010 world population estimates) live with some form of disability [7]. While different methodologies in the World Health Study and the Global Burden of Disease suggest slightly different prevalence estimates for adult disability, 15.6 and 19.4 %, respectively [7], both suggest that the global burden of disability is substantial. Similarly, in the United States, based upon data from the Survey of Income and Program Participation (SIPP), 18.7 % of non-institutionalized persons are living with a disability [8]. This number is expected to rise given the aging of the population [9, 10] and the high burden of chronic conditions including ischemic heart disease, stroke and HIV/AIDS [11].

Globally, women represent a rapidly growing proportion of the aging population given the projected increase in the life expectancy gender gap (reaching a gap of 4.4 years by 2050) in less developed countries [12]. The focus on contextual factors and their relevance for disability may be particularly important for the initiation of disability among women. The reported Male–female Health-Survival Paradox, whereby men have higher death rates but women fare worse in terms of disability and functioning [13–15] demonstrate that women are a particularly vulnerable group for disability problems as they age. The root causes of this paradox are unknown, but may include greater total disability burden among older women as compared to men [15], decreased likelihood of mortality among women with moderate to severe disability as compared to similarly-disabled men [15] or sex and gender differences in biological, behavioral and social factors across the lifespan. For example, research demonstrates that socioeconomic disadvantage is more strongly associated with disability risk among women as compared to men [16], and sex differences in body composition (higher total and subcutaneous fat mass and lower lean mass, muscle area and muscle density) translate to worse physical performance among women [17, 18] than among men. Among elderly populations, women experience more severe disability than men but it has been hypothesized that the combined impact of various social disadvantages such as lower income, less education, and higher prevalence of widowhood among older women may make them at greater risk for disability [19]. The male-to-female advantage in functioning is not, however, limited to elderly populations. Among mid-aged adults, women have 40 % lower levels of strength [20, 21], 20 % poorer balance times [20] and nearly twice the prevalence of self-reported difficulties with stair climbing activities [20] as compared to age-matched men. Further, women experience a more rapid decline in strength commencing in mid-life than do men; accelerations in strength loss begin between ages 40 and 55 in women whereas the loss of strength in men is linear across the lifespan [22–24]. The timing of this loss in strength has been noted to coincide with the timing of the menopausal transition [24–26] and some studies suggest that strength is preserved following menopause among women using exogenous hormone therapy [24, 25]. In addition to strength changes following menopause, data from cross-sectional studies show that postmenopausal women have 3.5 times higher odds of reporting substantial physical functioning limitations [26] and 17 % poorer balance times as compared to premenopausal women [27]. Given differences in functioning among pre- and post-menopausal women as well as among mid-aged men and women it has been hypothesized that ovarian function and the consequent decrease in estrogen levels during the menopausal transition may be associated with poor functioning.

### What is disability and how do we measure it?

**Fig. 1:**
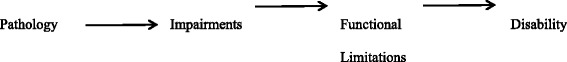
The disablement process by Verbrugge & Jette [17], adapted from Nagi [16]

The traditional model of disability and the disablement process was first described by Nagi [28] and then updated by Verbrugge & Jette [29]. As shown in Fig. 1, this traditional disablement model contributed substantially to our understanding by conceptualizing disability as a *process* during which one may experience impairments and limitations before reaching a disabled state. However, utilization of this model was limited by the focus on medical pathologies as the initiating factor in the cascade toward disablement. Although underlying medical conditions are known to be a risk factor for disability, a growing appreciation for the complexities underlying disability including the contextual and environmental factors prompted an international collaboration to revise and restructure this model.

**Fig. 2:**
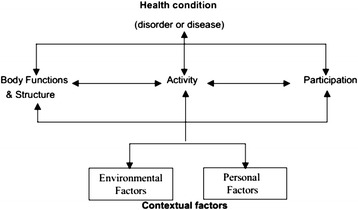
International Classification of Functioning disability framework

In 2001, the World Health Organization (WHO) officially endorsed the International Classification of Functioning (ICF), Disability and Health as the prevailing framework for measuring health and disability within individuals and populations. The ICF conceptualizes disability as a general construct not only defined by underlying pathology but by the interaction of individuals with their environment and the mediation of that relationship by underlying contextual factors including genetic, biological, behavioral, social and economic factors. This biopsychosocial model, shown in Fig. 2, is structured on three levels of functioning: body functions and structure, activity, and participation. Importantly, disability is not a condition of an individual but one that occurs for a given individual in certain contexts. Unlike earlier disability models, the ICF model includes both disease-related and non-disease-related disability, the latter of which may be particularly relevant among middle-aged populations who may or may not yet have manifested overt disease. Scientific interest in functional limitations and disability as health outcomes are motivated by the fact that declines in physical performance and the presence of disability are associated with increased risk of death, morbidity, and reduced quality of life [12, 16, 30]. Preservation of functioning and prevention of disability is critical so that individuals can maintain independence and remain autonomous as they age.

### Disability is an increasingly relevant mid-life health issue

While there has been a large focus on the impending “silver tsunami” given the known relationship between age and disability [8], evidence from five national studies suggests that activities of daily living (ADL) or instrumental activities of daily living (IADL) disability rates among elderly individuals have remained constant in recent years [31] and even show 0.61–0.90 % improvement per year in ADLs and 0.3–1.41 % improvement per year in IADLs among the oldest old (85+ years) [31].

Among mid-aged adults, however, an emerging body of literature suggests a remarkably high prevalence of disability during this life stage. The prevalence of mid-life disability has been reported to range from 20 to 40 % [5, 11, 32, 33] and most common types of disability are mobility-based [5, 32]. Most concerning, however, are the growing number of studies reporting temporal increases in disability among middle-aged populations, suggesting that this problem is becoming more exacerbated. Odds of ADL, IADL, and mobility disability were 1.3–1.7 times higher among 60–64 year olds in the 1999–2004 National Health and Nutrition Examination Survey (NHANES) as compared to the 1988–1994 NHANES [34], independent of obesity and chronic health conditions. Similarly, among 50–64 year olds in the National Health Interview Survey (NHIS), there was a 6.8–12.1 % increase in the number of individuals reporting difficulty with lower extremity mobility including difficulty stooping, standing for 2 h, walking a quarter-mile, and climbing ten steps without resting from the 1997–99 versus 2005–07 data collection cycles [33]. Among 40–64 years old in NHIS, the odds for physical functioning limitations, ADLs and IADLs increased annually by 0.9, 0.9, and 2.7 %, respectively; the increase in ADLs was independent of increases in obesity and was greater for women as compared to men [35]. In the Health and Retirement Survey (HRS), 15 % of adults aged 55–64 years in 2000 reported having difficulty with ADLs and there was a 0.1–0.2 percentage point increase per year [31]. However, the prevalence of ADL or IADL-assessed disability in mid-aged populations is relatively quite low as compared to older adults, and so more evidence is needed to confirm temporal trends and individual trajectories in disability.

With respect to physical functioning, the mid-life period is well accepted as a critical window for the onset of self-reported functional limitations [26, 36, 37] and diseases which ultimately lead to poor functioning and disability. In the Study of Women's Health Across the Nation (SWAN), a longitudinal study of midlife women, nearly one third of women (aged 45–57 years) reported moderate functional limitations and 11 % reported severe limitations based upon the SF-36 physical functioning questionnaire [26]. Similarly, in a British cohort of middle-aged adults, the prevalence of upper (difficulty gripping or reaching) and lower (difficulty walking or stair climbing) body limitations was 21–28 %, respectively [37]; the majority of these limitations began during the mid-life. Findings from NHIS show that deficits in functioning, defined as restricted activity resulting from illness, injury, or impairment begin during the mid-life [38] and that the most common conditions causing the need for help with ADLs or IADLs later in life, including back or neck problems, arthritis or rheumatism, diabetes, depression, anxiety, or emotional problems, hypertension, and nervous system conditions most commonly onset between 30 and 49 years of age [33].

Issues of functional limitations and disability are particularly salient among mid-aged individuals who are still in the work force and often caring for both dependent children and grandchildren as well as aging parents. Data from SIPP suggest that more than 10 % of mid-aged adults reported having limitation in their ability to work at a job [5]. Lack of employment during the midlife years may further compound health status and quality of life, as one’s health insurance and ability to pay for medical care is often tightly linked to their employment.

### Challenges to studying disability

While there is substantial interest in measuring and understanding functional limitations and disability, efforts are complicated by the multi-faceted nature of disability and the substantial diversity in the assessment methods used. To illustrate this point, please refer to Table 1 which was summarizes the variability in disability prevalence estimates and definitions used among studies reporting on mid-life disability. There is no consensus in the field as to how disability should be assessed or defined, and several different questionnaire and performance-based assessment tools are in operation, thereby resulting in highly variable prevalence estimates for disability. For example, two studies reporting disability rates among mid-aged women in Finland report wildly different estimates (7.8 % vs. 25.8 %) when using different definitions of disability [39, 40].

**Table 1:** Midlife disability prevalence and disability definitions

Reference	Study, year, geographic location	Midlife sample	Disability definition	Disability prevalence (95 % CI)
United States studies, national samples				
Altman & Gulley 2009 [45]	Joint Canada/United States Survey of Health, 2002–2003, United States and Canada national samples	40–64 years	Disability in 4 question domains: Restriction of Activities Screener (reduction of activities at home, school, work); Health Utilities Index (functional abilities including vision, hearing, speech, mobility, dexterity, emotional well being, cognition, pain); Activity and Participation Screener (restriction caused by physical, mental or emotional problem); and Physical Functioning Limitation	
		Men & women	• 40–49 years, Canada	19.82 % (14.16, 25.48)
			• 50–64 years, Canada	25.32 % (19.17, 31.47)
			• 40–49 years, United States	16.80 % (12.47, 21.13)
			• 50–64 years, United States	28.59 % (23.67, 33.51)
Mitra *et al.* 2009 [46]	Medical Expenditure Panel Survey, 2004, United States national sample	40–61 years	At least one of the following: limitations in work, housework, or school; walking limitations; cognitive limitation; limitations in seeing or hearing	
		Men and women	• 40–49 years	29.7 %
			• 50–61 years	45.4 %
Hottman *et al.* 2005 [5]	Survey of Income and Program Participation, 2005, United States national sample	45–64 years	At least one of the following: (women only)	25.9 %
		Men & women	• Use of an assistive aid	4.6 % (4.2, 5.0)
			• Difficulty performing ADLs	4.1 % (3.7, 4.5)
			• Difficulty performing IADLs	6.0 % (5.5, 6.5)
			• Difficulty performing specified functional activities	19.4 % (18.6, 20.2)
			• Reported of selected impairments	6.9 % (6.4, 7.4)
			• Limitation in ability to work around house	10.7 % (10.1, 11.3)
			• Limitation in ability to work at Job/business	11.3 % (10.7, 11.9)
Martin *et al.* 2010 [33]	National Health Interview Survey, 2005–2007, United States national sample	50–64 years	Difficulty with physical functions due to a health problem	42.0 %
		Men & women	• Needing help with IADLs	6.7 %
			• Needing help with ADLs	6.0 %
Zhao *et al.* 2009 [47]	Behavioral Risk Factor Surveillance System, 2005, United States national sample	50–65 years	Self-reported limitations in participation in activities because of physical, mental, or emotional problems or whether health problems required use of special equipment	
		Men & women	• 50–54 years	22.9 % (21.3, 24.5)
			• 55–59 years	28.8 % (27.8, 29.8)
			• 60–65 years	28.8 % (27.8, 29.8)
United States studies, local samples				
Khoury *et al.* 2013 [69]	Female Medicaid beneficiaries, 2001–2005, Florida	36–64 years	Presence of at least one physically disabling conditions but no use of a mobility assistive device	
		Women	• 36–45 years	35.79 %
			• 46–55 years	47.22 %
			• 56–64 years	53.30 %
			Presence of at least one physically disabling conditions and use of a mobility assistive device	
			• 36–45 years	2.92 %
			• 46–55 years	5.59 %
			• 56–64 years	9.17 %
Brown *et al.* 2014 [41]	Patients admitted to San Francisco General Hospital, 2010–2011, San Francisco, California	55–59 years	Needing help with at least one ADL 2 weeks before hospital admission	28.9 %
		Men & women	• Needing help with bathing	21.1 %
			• Needing help with dressing	20.5 %
			• Needing help with transferring	14.5 %
			• Needing help with eating	9.0 %
			• Needing help with toileting	9.6 %
			Needing help with at least 2 IADLs 2 weeks before hospital admission	36.1 %
			• Needing help with shopping	32.5 %
			• Needing help with light housework	30.6 %
			• Needing help with meal preparation	30.1 %
			• Needing help with transportation	21.1 %
			• Needing help with medication management	20.7 %
			• Needing help with money management	16.9 %
			• Needing help with using the telephone	7.2 %
Mann *et al.* 2015 [48]	Behavioral Risk Factor Surveillance System (BRFSS), 2011, South Carolina	45–64 years	Affirmative response to standard BRFSS disability questions:	
		Men & women	Self-reported limitation in “activities because of physical, mental, or emotional problems”	
			Or	
			Self-reported health problem that requires use of special equipment such as a cane, wheelchair, special bed, or special telephone.	
			• 45–54 years	22.1 % (20.0, 24.3)
			• 55–64 years	23.3 % (21.4, 25.2)
Karvonen-Gutierrez & Ylitalo 2013 [32]	Michigan Study of Women’s Health Across the Nation, 2011, Michigan	55.9–67.7 years	36-item World Health Organization Disability Assessment Schedule, severe-extreme disability:	
		Women only	• Global score	5.05 % (2.84, 7.26)
			• Understanding and communicating	5.05 % (2.84, 7.23)
			• Getting around	19.31 % (15.41, 23.41)
			• Self-care	4.26 % (2.22, 6.30)
			• Getting along with people	6.12 % (3.70, 8.54)
			• Engaging in life activities, non-work	43.16 % (35.07, 51.25)
			• Engaging in life activities, work	8.62 % (5.00, 12.23)
			• Participation in society	8.78 % (3.92, 11.64)
Arterburn *et al.* 2012 [52]	Group Health Plan enrollees, Washington	40–65 years	Modified World Health Organization Disability Assessment Schedule, any disability:	
		Women only	• Global score	Not reported
			• Understanding and communicating	26 %
			• Getting around	27 %
			• Self-care	7 %
			• Getting along with people	17 %
			• Engaging in life activities, non-work	46 %
			• Engaging in life activities, work	45 %
			• Participation in society	24 %
International studies				
Hosseinpoor *et al.* 2012 [16]	World Health Survey, 2002–2004, 57 countries	50–59 years	World Health Organization Report on Disability definition, based upon Item Response Theory model using data from questions in multiple domains.	
		Men & women	• 50–54 year old women	27.3 % (25.1, 29.5)
			• 55–59 year old women	30.5 % (28.0, 33.0)
Europe				
Kattainen *et al.* 2004 [39]	Finland Health 2000 Survey, 2000–2001, Finland	45–64 years	Blindness or being unable to perform without help or having marked difficulty at least one of	7.8 %
		Women	the following: moving about in the house, getting in/out of bed, dressing, carrying a 5-kg shopping bag, walking 500 m without rest, climbing a flight of stairs without rest, managing grocery shopping	
Krishnan *et al.* 2004 [40]	Cross-sectional study in Central Finland District, 2000, Finland	36–65	Health Assessment Questionnaire (HAQ) disability index score >0. HAQ assesses difficulty with performing activities in 8 functional categories: dressing/grooming, arising, eating, walking, hygiene, reach, grip, and common daily activities.	
		Women	• 36–40 years	14.7 % (7.9, 21.4)
			• 41–45 years	17.4 % (10.4, 24.4)
			• 46–50 years	25.0 % (17.4, 32.6)
			• 51–55 years	25.6 % (17.7, 33.5)
			• 56–60 years	36.7 % (27.0, 46.4)
			• 61–65 years	33.1 % (24.4, 41.7)
Klijs *et al.* 2011 [81]	Dutch PLOS-survey (Permanent Onderzoek Leefsituatie), 2001–2007, the Netherlands	55–59 years	Major difficulty doing or only able to do with help at least one of the following: walk up and down the stairs, walk outside, enter/leave the house, sit down/get up from a chair, move around on the same floor, get in/out of bed, eat/drink, get dressed/undressed, wash face/hands, wash completely	6 %
		Women		
Almazan-Isla *et al.* 2014 [60]	Residents from Cinco Villas, Spain, 2008–2009, Spain	50–59 years	36-item World Health Organization Disability Assessment Schedule, severe-extreme disability, women only	
		Men & women	• Global score	1.27 %
			• Understanding and communicating	1.27 %
			• Getting around	8.28 %
			• Self-care	2.55 %
			• Getting along with people	1.27 %
			• Engaging in life activities, non-work	12.74 %
			• Engaging in life activities, work	4.46 %
			• Participation in society	6.00 %
Africa				
Miszkurka *et al.* 2012 [54]	World Health Organization World Health Study, 2002–2003, Burkina Faso, Mali, and Senegal	35–64 years	Mobility disability, defined as self-reported mild, moderate, severe or extreme difficulty or unable to move around.	
		Men and women	• 35–44 years, Burkina Faso, women	21 % (16, 28)
			• 35–44 years, Mali, women	22 % (19, 26)
			• 35–44 years, Senegal, women	36 % (28, 44)
			• 45–54 years, Burkina Faso, women	25 % (19, 32)
			• 45–54 years, Mali, women	31 % (24, 39)
			• 45–54 years, Senegal, women	41 % (23, 63)
			• 55–64 years, Burkina Faso, women	52 % (40, 64)
			• 55–64 years, Mali, women	48 % (38, 58)
			• 55–64 years, Senegal, women	56 % (38, 72)
Payne *et al.* 2013 [55]	Malawi Longitudinal Study of Families and Health, 2010, Malawi	45–64 years	Having any health problem that limits ability to carry out culturally-relevant moderate activities or strenuous activities.	
		Men & women	• Moderately disabled (‘somewhat limited’ in either moderate or strenuous activities)	22.4 %
			• Severely disabled (‘limited a lot’ in either moderate or strenuous activities)	5.3 %
Wandera *et al.* 2014 [56]	Uganda National Household Survey, 2010, Uganda	50–59 years	Having a lot of difficulty or being unable to perform at least one of the following OR having some difficulty with at least two of the following: difficulty seeing, even if wearing glasses; difficulty hearing, even if wearing a hearing aid; difficulty walking or climbing steps; difficulty remembering or concentrating; difficulty washing all over or dressing, feeding and toileting; difficulty communicating because of a physical, mental or emotional health condition.	24.8 %
		Women		
Asia				
Zheng *et al.* 2011 [57]	China National Survey, 2006, China	45–64 years	Doctor-diagnosed disability following positive screen for self-reported visual, hearing, speech, physical, intellectual or mental disability	
		Men and women	• 45–54 years	11.0 %
			• 55–64 years	13.2 %
Peng *et al.* 2010 [58]	China National Sample Survey on Disability, 2006	35–64 years	Visual, intellectual, mental or physical disability assessed from an impairment-based examination	
		Women	• 35–39 years	3.48 % (3.41, 3.55)
			• 40–44 years	4.18 % (4.10, 4.26)
			• 45–49 years	5.32 % (5.21, 5.34)
			• 50–54 years	6.38 % (6.27, 6.49)
			• 55–59 years	8.77 % (8.62, 8.92)
			• 60–64 years	12.35 % (12.15, 12.55)
Hairi *et al.* 2010 [59]	Alor Gajah Older People Health Survey, 2007–2008, Malaysia	60–64 years	Level of independence in ADLs. 5-item scale included feeding, dressing, bathing, toileting and transferring. 6-item scale additionally included walking. 10-item scale additionally included grooming, bladder control, bowel control, and stair climbing.	
		Women	• 10 item ADL dependence	5.3 % (2.6, 10.1)
			• 6 item ADL dependence	4.7 % (2.2, 9.4)
			• 5 item ADL dependence	2.9 % (1.1, 7.9)

As shown in Table 1, many United States studies use either ADLs or IADLs as a disability measure. While these measures are relevant among elderly cohorts, the focus on self-care and ability to live independently may not be adequate to capture early deficits in functioning experienced by younger populations. As evidenced in Table 1, midlife disability prevalence estimates are lowest among studies using ADL or IADL definitions of disability among the general population where the prevalence ranges from 4.1 to 6.7 % [5, 33]. Notably, pre-admission ADL and IADL disability is much higher among a midlife sample of hospitalized patients (29–36 %) [41], suggesting the importance of ADLs and IADLs as a marker of poor health status.

Instead, studies among midlife populations often focus on physical functioning assessment with the assumption that deficits in physical functioning are a predictor of incident disability. The integrated nature of disability, rooted in the interaction between an individual and their environment, cannot be fully measured based upon physical functioning because variability in physical functioning does not full capture the full spectrum of limitations described by the ICF, particularly those that are contextual in nature and particularly relevant to mid-aged cohorts. For example, limitations in physical functioning may not lead to disability given one’s access to and use of adaptive strategies or resources. Further, one may be considered disabled for reasons other than limitations in physical functioning.

When physical functioning is used as a proxy for disability, it is assessed either based upon self-report using a variety of standardized and non-standardized questionnaires or based upon objective, performance-based measures which are often mobility-based. Evidence supports that self-reported and performance-based assessments measure distinct, yet related domains of physical functioning [42–44] but little work has been done to understand the correlation between physical functioning and ICF-based disability among community-based populations. Because physical functioning is only one aspect of an individual’s overall health and functioning, caution should be used when using physical functioning as a proxy for disability.

Many studies have considered self-reported limitation in (work, home, leisure, functioning) activities as a measure of disability. This paradigm is more closely aligned with the ICF framework by consideration of not only functioning but individual context. As shown in Table 1, disability prevalence using definitions based upon activity limitation are higher than those for ADL or IADL disability and increase by 40 % from early- to late-middle age in some [45, 46] but not all [47, 48] studies.

In many countries, work disability claims represent a potentially valuable resource for quantifying and studying the burden of disability among mid-life adults, as they are of working age. While such studies have contributed substantially to the literature and identified the importance of musculoskeletal functioning and mental health as major factors related to work-related disability, there are limitations in utilizing such databases for population research. First, work-related functioning and disability is often assessed in the context of one’s diagnosis and physical health and do not fully capture the impact of the psychosocial domains of disability [49]. Second, in occupational databases, there is often limited individual-level information about important causes, correlates, or consequences of disability such as that which is available from epidemiologic studies. This type of data is critical to identify potential strategies to prevent disability or to alleviate the individual burden of such limitations.

To support assessment of ICF-conceptualized disability, the WHO developed the Disability Assessment Schedule (WHO-DAS). The WHO-DAS questionnaire assesses disability in 6 domains including (a) understanding and communicating, (b) getting around, (c) self-care, (d) getting along with people, (e) engaging in life activities, and (f) participation in society, in addition to a global disability score. It is recognized and promoted as a universal and standardized measure of disability, suitable for national and international comparisons of disability prevalence and determinants across populations and age groups [50, 51]. While the WHO-DAS has been used to examine disability and its correlates in several clinical populations including those with mental health conditions, migraine, Parkinson’s Disease, multiple sclerosis, and traumatic brain injury, only two United States studies have examined WHO-DAS assessed disability in a general population of midlife adults. In the Michigan Study of Women’s Health Across the Nation (SWAN), WHO-DAS assessed disability prevalence was 25 % overall and at least 1 in 5 women reported moderate, severe or extreme problems with the understanding and communicating, getting around, getting along with people, work-related life activities and participation in society domains [32]. Data from a sample of women aged 40–65 years recruited from Group Health, a health insurance and care delivery system in the state of Washington, found that 45 % of women reported disabilities with work and non-work (i.e., household) activities and 27 % reported mobility disability [52]. Unlike studies among elderly cohorts [53] where the prevalence of self-care associated disability is nearly 40 %, only 1 in 10 midlife women in Michigan SWAN or the Group Health cohorts reported disability in the self-care domain [32, 52]. While WHO-DAS disability prevalence estimates (based upon the summary score) are similar to those published in the literature using other definitions of disability [5, 32, 33], the wide variation in domain-specific prevalence [33, 52] demonstrate the strength of the ICF framework in understanding the scope of disability during the mid-life.

Given national differences in medical care, support systems, and acceptability of aging, it is expected that international comparisons of disability prevalence would yield global variability. Unfortunately, cross-national comparisons of disability rates are complicated by variations in assessment method and definitions. As shown in Table 1, most disability work among midlife populations from Africa has focused on mobility disability or activity limitations and so prevalence rates range from 20 to 56 % [54–56]. While disability prevalence in Asia is appreciably lower (3–13 %), definitions are more conservative, based upon doctor diagnosis [57, 58] or ADL dependence [59]. The WHO-DAS has been used to assess disability among mid-life populations in the United States [32, 52] and Spain [60]. Disability prevalence rates were higher in the United States populations as compared to the Spanish population. However, the Spanish population included both men and women whereas the United States studies were among women only.

Differences in disability definitions, assessment strategies, and data sources can make it difficult to make comparisons between different studies, including national surveys, census-based data, and international agreements. Evaluation of trends in disability must be undertaken within longitudinal or panel studies using consistent measures and definitions. When synthesizing the literature and data regarding disability prevalence, incidence, and correlates, and particularly when making comparisons between studies, one must be careful to be cognizant of the constructs used to define disability.

### Functioning and disability are dynamic processes

Further complicating the consideration of disability among mid-life populations is that unlike elderly populations, mid-aged individuals may be more likely to experience disability due to acute, non-chronic events. Using data from NHIS of adults ≥18 years of age from 1988 to 2011, Iezzoni *et al.* [61] found a high proportion of non-chronic disability among respondents, ranging from 1 % for non-chronic social limitation disability to 40 % for non-chronic sensory difficulties. Similarly, data from SWAN has demonstrated that the presence of functional limitations during the mid-life is a highly dynamic process. While most SWAN women maintained their physical functioning level over a two-year period, 6–22 % of women worsened to a poorer level of functioning and 11–30 % of women actually improved their functioning [36]. Older adults also exhibit dynamic patterns of disability transitions, but unlike midlife populations, the vast majority exhibit worsening disability. In the Leiden 85-plus Study, a prospective cohort study of adults age 85 years and older, the prevalence of worsening disability after 5-years was 86 % [62], nearly 4 times greater than that among midlife women in SWAN [36]. Thus, the mid-life may be a highly malleable period during which interventions may be most efficacious because individuals may be more likely to have a propensity for improvement rather than deterioration. Consideration of the dynamic, non-chronic nature of disability status among mid-life populations is critical because most assessments are not designed to capture transient difficulties; estimates suggest that up to 40 % of disability complaints are missed among mid-aged populations because discordance between measurement window and timing of disability [61]. Thus, in mid-aged populations, repeated assessment and data collection is critical to fully understand the burden of disability during this life stage.

### Improving disability among mid-life women

Mid-life factors including stress, low social support, decreased social activity, physical inactivity, poor physical functioning, smoking, obesity and diabetes [63–65] are known to predict old age disability. The high prevalence of disability during the mid-life period [5, 11, 32, 33], however, raises the urgent need to intervene to prevent not only future disability but also present disability. The mid-life period is a time of dynamic changes in physical functioning and mid-aged individuals have a high capacity for improvement [36], so there is an imperative need to understand correlates of mid-age disability so that we may develop appropriate and efficacious interventions. Correlates of mid-age disability include obesity [52, 66, 67], depression symptoms [32, 52, 68], economic strain [32] and chronic disease comorbidity [69] and burden [47] including knee osteoarthritis and peripheral neuropathy [32]. Further, incident disability later in life is predicted by mid-life depression [70], increased body mass index [70–72], poor physical functioning performance [68, 73], low levels of physical activity [71, 73] and smoking [72].

As chronic conditions are the leading causes of disability in the United States [5] and globally [6], efforts to prevent disease or reducing symptomatology at earlier ages is one critical strategy to prevent or forestall disability. Many of the conditions which are major correlates of disability emerge or are more bothersome during the mid-life, including osteoarthritis [74], heart trouble [75], low back pain [76] and mental and emotional health problems [77] and are further exacerbated by obesity [78–80]. Among mid-life women, arthritis and back pain have the largest contribution to disability prevalence [81]. Thus, while much work has been done to intervene on disease-specific conditions in older adults as an effort to improve functioning and reduce disability, evidence suggests that interventions starting in midlife or earlier may be most beneficial in reducing disability risk among both midlife and older adults [47].

Individuals may be most amenable to interventions during the mid-life, as evidenced by the success of ergonomic, vocational rehabilitation, and strength training work-place interventions shown to reduce back and upper limb pain-associated work disability [82–84]. However, there has been a dearth of intervention studies among mid-life adults beyond work-place interventions to reduce sick leave or work-related disability. One potential reason for this is the belief that a prohibitively long follow-up period will be needed to observe any effects of an intervention. While recommendations for high-impact interventions for disability reductions among late-life adults have been published [85], no such statement has been issued for mid-life adults. However, given the high prevalence of disability and functional limitations among mid-life populations, this concern is mitigated by the opportunity to improve functioning and disability *during* the midlife. Given increasing trends in disability prevalence [31] and chronic conditions [2, 3] among mid-life adults, efforts to improve the health and functioning during this life stage is highly needed to appropriately address the unique health needs this population. Additionally, to fully understand how to *prevent* disability and improve health for late-life individuals, we must identify interventions that, when implemented early, have the ability for sustained benefit as one ages.

Studies among older adults, however, do provide insight to interventions that may be efficacious among mid-life populations. Multi-component exercise interventions [86] including the Lifestyle Interventions and Independence for Elders (LIFE) study [87, 88] and interventions to reduce depression symptoms [89] have showed promising results in reducing incident disability among older adults. Depressive symptoms and decreased physical activity are predictors of mid-life incident disability. This knowledge – the utility of depression and physical activity interventions among older adults and the importance of these factors for predicting mid-life disability suggest that they may be relevant areas for mid-life intervention studies. Further, a simulation study using data from the Nurses’ Health Study suggest that midlife weight loss and physical activity interventions would be most efficacious in preventing chronic disease incidence, reducing risk by up to 10 percentage points [90]. The current pressing challenge, however, is to develop and implement interventions that, when begun during the mid-life, have the capacity for long-term adherence and effectiveness so as to impact long-term health, functioning and wellness trajectories.

Another challenge to intervention studies among mid-life populations is the highly dynamic nature of mid-life functioning and disability, thereby signaling a need for different frameworks and interventions to impact the onset and recovery from functional limitations and disability. In HRS among adults age 51–61 years, recovery from mobility disability over 2 years was predicted by lack of diabetes, lung disease and pain whereas onset of mobility disability was predicted by being female, less educated, obese, and having frequent pain [91]. Among women SWAN, highly dynamic patterns of functioning, characterized as both worsening and improving over time, were observed among obese women and women who had arthritis [36]. Therefore, different intervention programs and paradigms may need to be considered which target mid-life factors to prevent old age disability versus those that can prompt recovery from current mid-life disability.

## Conclusion

Disability prevalence is high during the mid-life, yet domains of disability among younger populations differ substantially from those among older adults. Despite a high burden, evidence suggests that the presence of mid-life disability does not inevitably worsen. Instead, encouraging data suggests that mid-aged individuals are highly capable of recovering from non-chronic disability. This observation, combined with the known detrimental effect of poor functioning and disability on current and further health should prompt a concentrated public health effort to target interventions to improve functioning and prevent disability among mid-aged adults. Focused efforts on treatment of depression and physical activity interventions to reduce obesity and prevent mobility disability may be most efficacious during this life stage.

## Abbreviations

SIPP: Survey of Income and Program Participation
WHO: World Health Organization
ICF: International Classification of Functioning
ADL: Activities of daily living
IADL: Instrumental activities of daily living
WHO-DAS: World Health Organization Disability Assessment Schedule
SWAN: Study of Women’s Health Across the Nation
NHANES: National Health and Nutrition Examination Survey
NHIS: National Health Interview Survey
HRS: Health and Retirement Survey
BRFSS: Behavioral Risk Factor Surveillance System
